# Measuring the impact of COVID‐19 on stock prices and profits in the food supply chain

**DOI:** 10.1002/agr.21678

**Published:** 2020-12-09

**Authors:** Julia Höhler, Alfons Oude Lansink

**Affiliations:** ^1^ Business Economics Group Wageningen University Wageningen Netherlands

## Abstract

The coronavirus disease 2019 pandemic has shocked financial and commodity markets around the world. We are analyzing stock prices and information from financial reports to examine the impact of the pandemic on stock price volatility and profits of companies in the food supply chain. We use a data set of 71 major listed companies in the food value chain from stock indices in the US, Japan, and Europe. We calculate the annualized volatility per sector, screen the contents of the reports for stated effects of the pandemic on profits, and analyze stock price reactions in four different phases of the pandemic. The results show that stock markets have reacted with an increased price volatility. Manufacturers of fertilizers and agrochemicals as well as food distributors show particularly high volatilities in their stock prices. Low price volatility was observed in the stocks of food retailers. This pattern is also reflected in the profits of companies published in financial reports. Our regression analyses indicate that stocks of more profitable companies exhibited higher cumulative returns during the outbreak. In the phases thereafter, riskier stocks received higher discounts on returns. EconLit citations: G01, G12, E44, Q01.

## 1 INTRODUCTION

The outbreak of coronavirus disease 2019 (COVID‐19) as of early 2020 has had a massive impact on the economy around the globe (Baldwin & Tomiura, 2020). This also applies to the companies in the food supply chain, who are affected in different ways (e.g., Gray, 2020; Hailu, 2020). Newspapers report about the lack of harvest workers in agriculture, slowed distribution, massive outbreaks among personnel in slaughterhouses and empty shelves in grocery stores (Financial Times, 2020; New York Times, 2020). All these effects imposed significant costs on the companies concerned and reduced food security (Gundersen et al., 2020). Government interventions have aimed at mitigating the impact of the COVID‐19 crisis on the value chain (Reuters, 2020a). At the same time, the exact consequences are often subject to continuing uncertainty (Baker et al., 2020). The OECD expects serious losses in economic growth and reduced consumer spending, particularly if the outbreak persists over a longer period. A deeper understanding of the effects of the crisis is necessary, particularly in view of the debt situation of many companies. Volatile markets can lead to a sharp increase in businesses' financing costs and may threaten the liquidity and solvency, and ultimately the continuity of companies (OECD, 2020a).

One way to investigate possible effects of the pandemic on businesses is to analyze stock prices (see also Ramelli & Wagner, 2020). In contrast to other data sources, they allow for the estimation of the consequences of an event without long observation periods (Estrella & Mishkin, 1998; MacKinlay, 1997). According to the theory of efficient markets, stock prices represent the expected present discounted value of dividends (Fama, 1970; West, 1988). Changes in stock prices are the result of changing expectations about future returns and risks. Information on stock prices and their volatility is relevant from the perspective of both managers and shareholders (Wang et al., 2002). For the 2008 financial crisis, it has been shown that effects on stock prices are also reflected in firm performance (Claessens et al., 2012). In addition to stock prices, financial reports are of major importance for the functioning of efficient markets. They serve shareholders, analysts, and potential investors as a source of information on the current and expected future performance of companies (Healy & Palepu, 2001). For the beginning of the COVID‐19 pandemic, stock prices and (quarterly) reports provide information on how and to what extent companies were affected by the outbreak according to investors. Initial investigations show that returns in the sectors “food and staples retailing” and “food, beverages, and tobacco” were less affected than in other sectors (Ramelli & Wagner, 2020). However, it is unclear how individual sub‐sectors and stages of the food supply chain were impacted (Nayga & Zilberman, 2020).

Previous studies of stock price returns during COVID‐19 have not dealt with the peculiarities of the food sector which differs in terms of its susceptibility to shocks from other sectors. Farm products are often bulky and perishable, which places significant constraints on geographical mobility and increases transport costs (Rogers & Sexton, 1994). In addition, some companies in the food supply chain heavily rely on human labor for activities like harvesting, processing and transportation. COVID‐19 has caused a supply shock due to reduced mobility of workers, delays in transportation and limited availability of some inputs. At the same time, it has triggered a demand shock by shifting the composition and level of demand for food (e.g., less out‐of‐home consumption). These simultaneous shocks can affect both food availability and prices, to the point of threatening the food security of individual households (OECD, 2020b). We hypothesize that COVID‐19 and the resulting shocks have affected companies in the value chain in opposite ways. The temporary closure of many out‐of‐home food outlets has led to people stocking up on nonperishable food. It can therefore be assumed that food processors and retailers have benefited. Conversely, food distributors have lost some of their marketing channels, so that losses in this sub‐sector are more likely. For other sub‐sectors, such as soft drinks or alcoholic beverages, the impact is less clear, as the reduced consumption out‐of‐home may not have been compensated by increased purchases. A differentiated analysis of the impact on different sub‐sectors is necessary to identify possible weaknesses in the food supply chain.

Evidence suggests that shareholders can use agribusiness stocks to diversify their portfolio and thus minimize risk (Clark et al., 2012; Katchova & Enlow, 2013).^1^ However, there is a lack of knowledge about how agribusiness stocks react to shocks compared to the general market (Clark et al., 2012). Previous research on the effect of shocks on agribusiness has focused on how agribusiness companies in single countries are affected by shocks within the sector, such as the BSE crisis or the foot‐and‐mouth disease outbreak (Jin & Kim, 2008; Pendell & Cho, 2013), rather than the effect of global pandemics. In the aftermath of the severe acute respiratory syndrome (SARS) outbreak, Chen et al. (2007) analyze the impact of SARS on stock performance in Taiwan with a focus on the tourist industry. For the food industry, they report negative abnormal returns in the 10 days after the outbreak, but not in the 20 days after the outset of the outbreak. However, the results cannot be transferred to SARS‐COVID‐19, since the higher reproductive rate has already led to a much wider geographical spread and higher death rates (Liu et al., 2020; Zhang et al., 2020). The current COVID‐19 crisis is unique in its “sudden and disruptive nature” (Ramelli & Wagner, 2020, p. 626).

Many researchers have utilized event studies to measure the effect of shocks on stock market prices. In their study of the impact of SARS on Taiwanese hotel stock performance, Chen et al. (2007) use an event‐study approach to analyze abnormal returns. Wang et al. (2002) employ a GARCH model to investigate the stock market reaction to food recalls. Event studies relate the stock price development of a sample or firm to the return of an underlying index or portfolio. However, since the COVID‐19 also affects index values, it is difficult to separate the effects of the pandemic from market effects. Furthermore, it is unclear what the event date and event window should be, as the situation is characterized by a series of news (Ramelli & Wagner, 2020). McWilliams and Siegel (1997) argue that long event windows are not compatible with the assumption of efficient markets. However, information on infectivity and the extent of the crisis only became known over time (Del Rio & Malani, 2020). It can be assumed that they were only factored into stock prices over time. Therefore, we evaluate the development of stock prices over different phases of the pandemic.

The main aim of this study is to investigate the impact of the COVID‐19 pandemic on the volatility of stock prices and on profits of a sample of stock listed companies in the food supply chain. The research data is drawn from two main sources: Stock exchange data and financial reports. We employ a broad definition of “food supply chain” that covers the entire supply chain from farm equipment and supplies, agriculture, trade, processing, distribution to retailing. The 71 companies investigated are located either in the USA, Europe, or Japan. In a first step, we analyze their stock prices by calculating the annualized volatility and comparing it across industries and companies. This data enables us to compare the situation with other shocks such as the 2008 financial crisis. It also allows to identify which sub‐sectors were particularly affected. To cross‐validate our results, we systematically evaluate financial reports for each company. Furthermore, we run regressions to analyze the impact of company characteristics on cumulative returns in four different phases of the pandemic. In this way we show which company characteristics have influenced the susceptibility to the COVID‐19 induced shock. The results are intended to provide first insights into the effects of COVID‐19 on companies in the food supply chain.

There are several areas where this study makes an original contribution to. First, we contribute to the understanding of the impact of shocks on the volatility of stock prices and profits. Second, we contribute to the literature in the field of research on stock market prices of agribusiness companies by being the first study to compare data from three continents. To date, there is little evidence on the impact of the COVID‐19 pandemic on the food supply chain. Our results can be helpful for managers who are preparing for the next crisis by showing which companies were particularly affected and benchmarking different sectors. An improved understanding of the impact of the pandemic can also help in the design of policy measures to support companies.

The remaining part of the paper proceeds as follows: We first present the methods for measuring and analyzing stock returns, volatility and profits. We then describe the data set. This is followed by the presentation of the results. We end the paper with a discussion and conclusions.

## 2 MEASURING AND ANALYZING STOCK RETURNS, VOLATILITY, AND PROFITS

The capital asset pricing model (CAPM) explains expected returns on risky assets as the result of a risk‐free interest rate, expected returns of the market and a beta factor. The beta factor measures the risk of an asset in comparison to the market index (Lintner, 1965; Sharpe, 1964). Betas greater (less) than one should therefore result in higher (lower) returns compared to the average of the market. Based on various empirical studies that rejected the CAPM, Fama and French (1992) developed their Fama‐French three factor model. In their model, they assume that not only high risks but also low market capitalization and high book‐to‐market values are associated with higher returns. In 2015, they extended their model by profitability and investment factors (Fama & French, 2015). Empirical evidence suggests that the effects of the factors are country‐specific (Fama & French, 2017; Griffin, 2002); also the empirical evidence shows that periods of crisis shift the relationship between factors and returns (Zhang, 2005).

High levels of market volatility are associated with negative stock returns and high price risk premiums (Banerjee et al., 2007). Let *r*_j,t_ be the percentage change in the stock price of stock *j* from day to day (i.e., the day to day return):

$$r_{j,t}=\ln\left(\frac{P_{j,t}}{P_{j,t-1}},)\right)\times 100,$$

where *P*_j,t_ is the adjusted closing price of stock *j* on day *t* and *P*_j,t−1_ is the adjusted closing price of stock *j* on the previous day. The volatility of *r*_j,t_ (*σ*_r_) can be calculated using the usual standard deviation formula. To establish a comparability of different time periods, the daily continuous standard deviation is multiplied by the square root of 252 (assuming 252 trading days) to get the annualized continuous volatility (e.g., Moles & Terry, 1997):

$$\sigma_{\mathrm{annualy}}=\sigma_{r}\times \sqrt{252}.$$

For the analysis of the impact of the pandemic on stock prices, we follow the approach of Ramelli and Wagner (2020). The authors use data on cumulative raw returns (and CAPM‐adjusted cumulative returns) of Russell 3000 constituents as dependent variables and estimate three basic models for three different phases of the pandemic. Ramelli and Wagner (2020) base the phases on trading days in the United States and specify them as:

(1) Incubation, January 2–17: First notification of COVID‐19 to the WHO; Chinese health authorities closed down Huanan Seafood Wholesale Market.
(2) Outbreak, January 20–February 21: Chinese health authorities confirmed the human‐to‐human transmission; WHO published the first situation report on the outbreak.
(3) Fever, February 24–March 20: First deaths from coronavirus in Italy. Subsequently, further outbreaks and lockdowns around the world.

In their model, the beta factor of 2019 serves as an independent variable, along with other company characteristics (market capitalization, profitability, and book‐to‐market value) for the purpose of control. In contrast to their model, we do not take into account exposure to China and foreign revenues. In their study, they included the number of times China is mentioned in a company's 10‐K report. The different formats of the reports we examined do not allow a comparison of this key figure. Also, information about foreign revenues is not available for most of the companies in our data set. Also, unlike the study of Ramelli and Wagner, the companies in our sample are not only based in the US but in different financial systems (see also Semenov, 2006); for that reason we control for country effects (Japan and US). We assume that the selected phases also apply similarly to the other regions, as they largely refer to general and not to country‐specific events. As more information on the course of the pandemic has been added in the meantime, we are extending the model to include an additional below peak phase. The phase runs from March 23–April 29. The length of the period was chosen to allow for comparability with the previous phases. In addition, on March 23 the Federal Reserve Board announced interventions in the market for corporate bonds. Since then, many other countries have also passed aid packages to relieve the financial pressure on companies.

## 3 DATA SET

Our data set includes stock price information of companies in different stock indices, whereby the S&P 500 is the largest index. The S&P 500 measures the stock performance of 500 large companies listed in the United States. To be included in the index, companies must have an unadjusted market capitalization of 8.2 USD billion or more (Standard & Poor's, 2020). Other indices used are the British FTSE, the German DAX, the French CAC, the Belgian BEL, the Dutch AEX, the Swiss SMI, and the Japanese Nikkei index. Indices were chosen because they account for a large share of the market capitalization in the respective markets. The selection was made according to various criteria: First, it was important to cover different regions. As each of the European indices covers relatively few companies, several indices were used. Second, the selection was based on whether companies from the food supply chain were listed in the index. The included stock indices use various industry classifications. Companies in the S&P 500 are classified based on the Global Industry Classification Standard (Standard & Poor's Global Market Intelligence, 2018). As this is the largest index in our data set, we used the classification and recoded the companies of other indices accordingly. This was done, in particular for the Nikkei index, using the information about the companies on the Reuters news platform (Reuters, 2020b).

Stock prices were retrieved from Yahoo! Finance using the tidyquant package (Dancho & Vaughan, 2020). The stock prices were collected over the period 2000–2020. The rvest package was used to scrape the relevant symbols from Wikipedia (Wickham, 2019). The combination of the two data frames generates a list of stock prices for each company. The output includes date, open price, high price, low price, close price, volume, and adjusted price. Exchange rates from Yahoo! finance, and, due to the unavailability of pre‐2003 exchange rates, from Investing. com, were used to convert all stock prices to US dollars. The data was adjusted for implausible values (negative prices or unexplainable price jumps of more than 100 percent). In this context, one company was removed from the data set as it repeatedly exhibited negative prices and large price jumps. The final data set includes 71 companies from 11 sectors in the food supply chain (Table 1). Most of the companies (30) are located in the US, 21 on the European continent and 20 in Japan (for a detailed overview, see Appendix A).

**Table 1:** Distribution of companies in the data set by sector and region

**Sector**	***n***	**US**	**Japan**	**Europe**
Packaged foods and meats	23	12	6	5
Food retail	13	1	5	7
Brewers	7	1	4	2
Fertilizers and agricultural chemicals	7	4	1	2
Tobacco	4	2	‐	2
Soft drinks	4	3	1	‐
Distillers and vintners	4	2	‐	2
Food distributors	3	1	2	‐
Agricultural and farm machinery	2	1	1	‐
Hypermarkets and super centers	2	2	‐	‐
Agricultural products	2	1	1	‐
Total	71	30	20	21

*Source*: Own illustration

Due to a different number of trading days on the various stock exchanges, an average of 4770.4 trading days per company was observed. For 2020, 81.2 trading days per company were observed.

The next step was to collect data from the financial reports of the companies. This was done via the respective websites for investor relations on June 22, 2020. In total, financial reports for 67 companies could be found. Due to different legal frameworks, the reports differ in terms of their scope, focus and the period covered. They are either annual reports, quarterly reports or trading statements. All of them cover months of the year 2020 and 60 of them contain information about operating profits, 48 of them include March 2020, 41 of them are quarterly reports. The change in profits compared to the previous period was calculated from the reports. In addition, statements about the outlook were collected from the reports.

For the regression model, additional financial data on the companies were obtained from the Orbis platform (Bureau van Dijk, 2020). As data for 2019 were only available for 56 companies, additional data for 2018 and 2019 were collected. Based on the data, book‐to‐market was calculated as the inverse price book value ratio—average high‐low. A logarithmic transformation was applied to the variable market capitalization. Profitability was calculated as return on assets in terms of net income over total assets (in %). Leverage was calculated as the percentage of long‐term debt in total assets. Cash‐to‐assets was calculated as percentage of cash and short‐term investments in total assets. The market beta was calculated by means of a linear regression and based on S&P500 returns in 2019 (Groenewold & Fraser, 1999). Due to the limited sample size, no subsector‐specific effects were estimated in the model.

Table 2 summarizes the descriptive statistics for variables used in the regression analysis. Due to the missing values in 2019, the financial data (i.e., the independent variables) are those of 2018. The only exception is beta. Since one of the companies did not exist in 2018, complete data is only available for 2019. In addition, there is a strong correlation (0.75) between the 2018 and 2019 beta values of the other companies.

**Table 2:** Descriptive statistics of regression variables

**Variable**	**Mean**	**Min**	**Max**	**Standard deviation**
Dependent variables				
Percentage change in stock price (*r*)				
2019	12.8	−29.1	55.8	19.9
Incubation phase (2020)	1.2	−8.3	8.0	3.8
Outbreak phase (2020)	−2.6	−18.5	11.5	6.8
Fever phase (2020)	−25.2	−76.0	8.5	19.2
Below peak phase (2020)	−14.2	−56.4	20.1	16.3
Independent variables (2018)				
Book‐to‐market	0.48	−0.1	1.4	0.4
Market capitalization (log)	9.9	7.4	12.5	1.15
Profitability	6.2	−9.9	36.1	6.4
Leverage	25.4	0	84.9	14.9
Cash‐to‐assets	6.7	0	30.4	6.7
Beta 2019	0.49	0	1.5	0.3

*Note*: Excluding dummy variables Japan and US.

## 4 RESULTS

**Figure 1:**
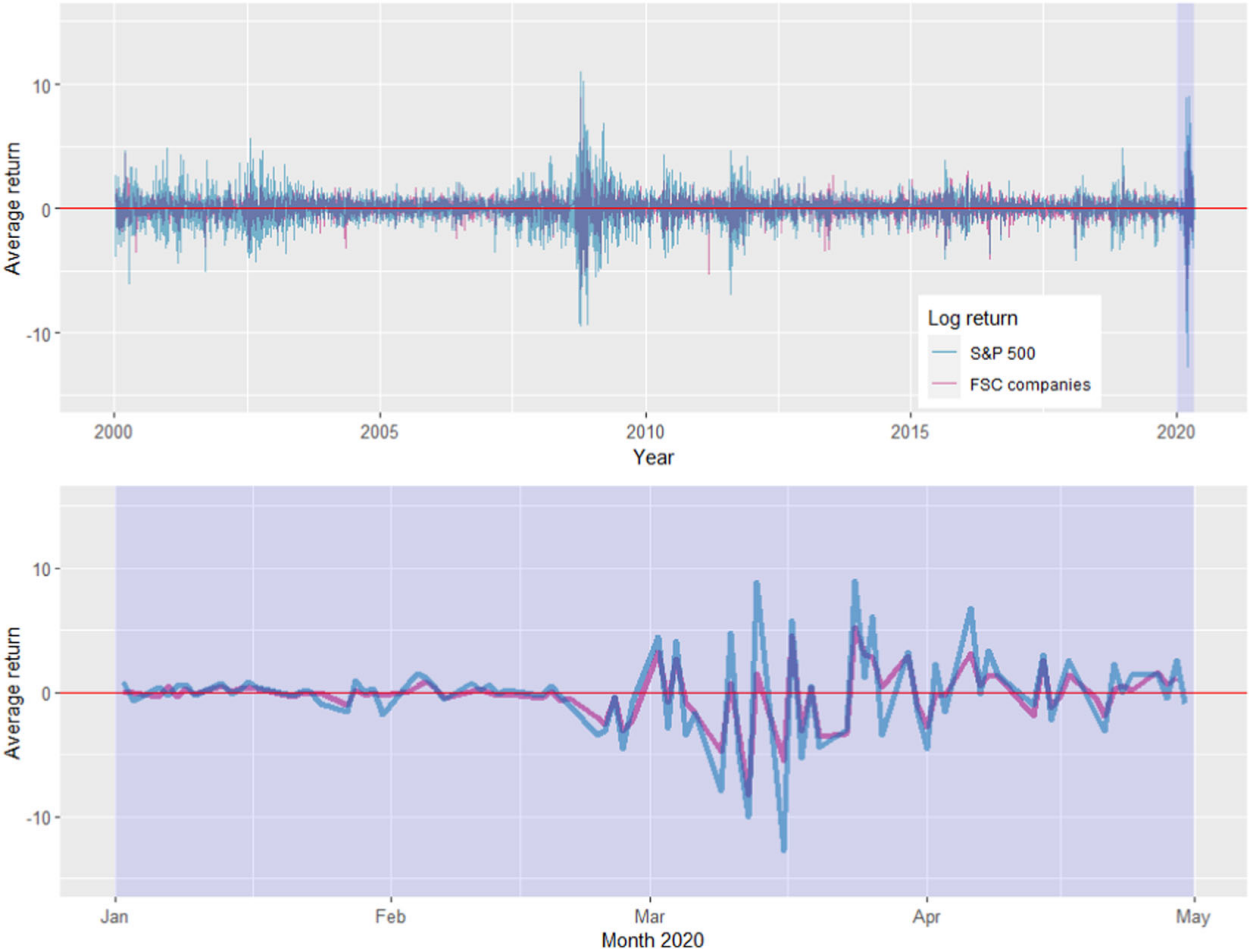
Average daily returns 2000–2020 and January–April 2020. *Source*: Own illustration [Color figure can be viewed at wileyonlinelibrary.com]

Figure 1 shows the average daily returns from 2000 to May 2020, and from January to May 2020 for both the S&P 500 and our data set (FSC companies).

High fluctuations are evident in the context of the financial crisis of 2008. The period covering the COVID‐19 pandemic is also characterized by an above average volatility. The negative returns exceed those of the 2008 financial crisis slightly, while the peak in the positive returns is lower. The chart in the bottom section shows that on average, the FSC companies had fluctuations in returns that were slightly lower than those of the firms in the S&P500.

### 4.1 Volatility

**Figure 2:**
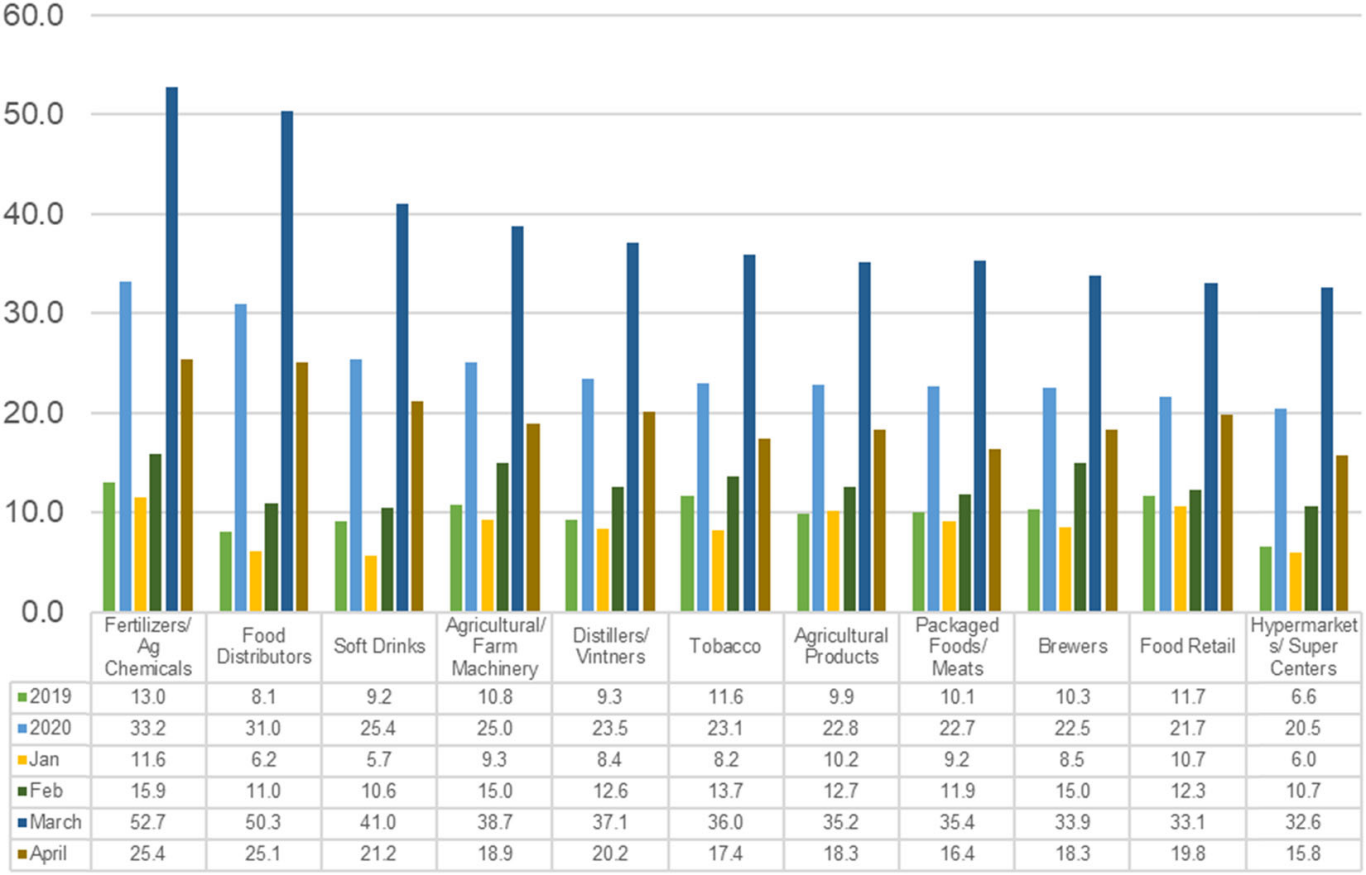
Annualized volatilities 2019, 2020 as well as January–April 2020. *Source*: Own illustration [Color figure can be viewed at wileyonlinelibrary.com]

Figure 2 shows the annualized volatilities per sector in the years 2019 and 2020 as well as in the months January to April 2020. Overall, volatility has increased in 2020, with a peak in March. The highest volatility was observed in the fertilizer and agrochemicals subsector, followed by food distributors. Among food distributors and soft drink companies, volatility was among the lowest of the sub‐sectors in 2019 and among the highest volatilities in 2020. Comparatively low volatility was found in food retail as well as hypermarkets and super centers. The food retail sector recorded the lowest overall increase in volatility from 2019 to 2020, which is in line with the observation that the retail sector did not suffer in the early months of the COVID‐19 outbreak.

### 4.2 Profits

**Figure 3:**
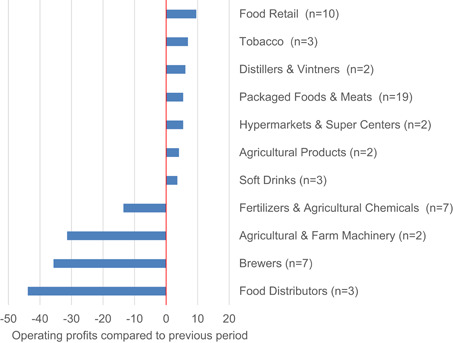
Operating profits per sector compared to previous reported period. *Source*: Own illustration [Color figure can be viewed at wileyonlinelibrary.com]

Figure 3 shows the change of operating profits compared to the previous period (quarter or year). While the three food distributors in our sample recorded average losses in operating profits of 43.9 percent, food retailers were able to increase their profits by 9.6 percent. In the case of the breweries, there were differences between the stock price development and the reported operating profits. While the companies recorded average decreases of 35.7 percent, their stock prices seemed less volatile compared to the other sectors.

The information collected on the outlook from the financial reports of the companies shows that some companies have withdrawn their outlook. Frequently it was replaced by statements like the following: “*given the global nature of the COVID‐19 pandemic, and the uncertainty around the severity and duration of the impact across multiple markets, we are not in a position to accurately assess the impact of this on our future financial performance. We are therefore withdrawing our guidance on group organic net sales growth and organic operating profit*” (Diageo, 2020).

### 4.3 Stock returns

The following table (Table 3) shows the models for the four different phases and for 2019. Variance inflation factors (VIF) were calculated to test for multicollinearity. The VIF scores below 10 indicate that multicollinearity is not inflating the standard errors. The average VIF score was 1.8.

**Table 3:** Cumulative stock returns in 2019 and during four phases of the pandemic

Dependent variable: Cumulative returns					
		Incubation	Outbreak	Fever	Below peak
January 02–17	Year 2019	January 02–17	January 20–February 21	February 24–March 20	March 23–April 29
	(1)	(2)	(3)	(4)	(5)
Beta	5.551	−0.022	−3.389	−33.374***	−24.097***
	(8.485)	(1.658)	(3.050)	(8.189)	(6.918)
Log market capitalization	1.045	−0.002	−0.182	0.934	1.323*
	(0.869)	(0.170)	(0.313)	(0.839)	(0.709)
Profitability	0.064	0.049	0.249*	−0.634	−0.588*
	(0.399)	(0.078)	(0.144)	(0.385)	(0.326)
Book‐to‐market	−11.058	−3.846**	−1.369	−3.721	−11.766*
	(8.095)	(1.582)	(2.910)	(7.813)	(6.600)
Cash/assets	−0.142	0.121*	0.098	0.061	0.189
	(0.352)	(0.069)	(0.126)	(0.339)	(0.287)
Leverage	0.272	0.031	−0.019	−0.490***	−0.337**
	(0.164)	(0.032)	(0.059)	(0.159)	(0.134)
Dummy Japan	−7.412	2.656*	−3.480	−8.250	−10.663*
	(6.888)	(1.346)	(2.476)	(6.648)	(5.615)
Dummy US	2.072	1.088	1.313	5.135	9.088*
	(5.614)	(1.097)	(2.018)	(5.418)	(4.577)
Observations	70	70	70	70	70
*R* ^2^	0.520	0.336	0.313	0.748	0.619

*Note*: One company was excluded due to missing values.

* *p* < .1.

** *p* < .05.

*** *p* < .01.

The results show that the company characteristics contribute differently to the explanation of the cumulative returns in the four phases. In the incubation phase, undervalued stocks, that is, stocks with a book‐to‐market ratio above 1, had lower cumulative returns. During the same phase, more liquid companies (higher cash‐to‐assets ratio) and Japanese companies were able to achieve higher cumulative returns. Compared to Europe, the cumulative returns of Japanese companies' stocks were, on average, 2.7 percentage points higher.

In the outbreak phase, higher profitability was associated with higher cumulative returns. For example, an increase in profitability of 10 percentage points was accompanied by a 2.5 percentage point higher cumulative return. In the fever phase, higher betas and leverage were statistically significantly associated with lower returns. Under otherwise unchanged conditions, an increase of beta by 0.1 points is associated with a 3.3 percent lower cumulative returns. An increase of the leverage, that is, cash and short‐term investments over total assets, by 10 percentage points is associated with a decrease of the cumulative returns by 4.9 percentage points. In the phase below the peak, the coefficients of beta, profitability, book‐to‐market value and leverage indicate a statistically significant negative association with returns. The dummy variables suggest differences in stock price reactions between the regions. Cumulative returns in the below peak phase were higher for Japanese and lower for US stocks compared to stocks of European companies.

In the first two phases of the pandemic, it is also notable that the model has a lower explanatory power. In addition to the models in Table 3 and to check the robustness, models with 2019 company data were estimated (*n* = 56, see Appendix B). Most signs remain unchanged. The statistical significance remains the same for betas and leverage, while it changes for some other variables. The biggest changes in coefficients are in the book‐to‐market ratio, which in the 2019 model has a positive (but statistically not significant) effect on cumulative returns in the last two phases.

## 5 CONCLUSION AND DISCUSSION

The purpose of the current study was to provide an initial assessment of the impact of COVID‐19 on the volatility of stock prices and profits of companies in the food supply chain. To achieve this, various data sources and calculations were combined. First, stock price returns of large stock‐listed companies over the period 2000–2020 in the food supply chain were calculated and compared. Overall, stock price volatility was lower than in the S&P500 which supports the conclusion of Ramelli and Wagner (2020) that the food sector was less affected by COVID‐19 than other sectors. It is also in line with previous research on the volatility of agribusiness stocks compared to the S&P 500 (Clark et al., 2012). A comparison with other shocks such as the 2008 financial crisis can help to approximate the severity of the shock caused by COVID‐19. Our analysis shows that the downward swings in returns during the first four months of 2020 were slightly larger. Although the two shocks differ in their causes, the financial crisis provides indications of possible further consequences of COVID‐19. These include an increase in insolvencies, rising unemployment, and continued food insecurity (Baldwin & Tomiura, 2020; Gundersen et al., 2020).

High levels of stock price volatility are associated with increases in financing costs and high price risk premiums. The highest stock price volatility during the beginning of the pandemic was in the fertilizer and agrochemicals subsector. A possible explanation for this higher volatility might be the industry's dependence on oil prices. The food distributors also showed comparatively high volatilities. Reasons for this could be the lockdowns and social distancing measures which resulted in a lack of sales opportunities, especially for products such as seafood or dairy products (OECD, 2020b). The cross‐validation with data from the financial reports confirmed that both sub‐sectors have also recorded negative operating profits. For both sectors, however, only small sample sizes were available, so that our conclusions can only be considered as a first indication. Food retailers and producers of packaged food showed low stock price volatility compared to the other sectors. This is also reflected in the operating profits, which have increased compared to the previous reference period. Overall, our results support the hypothesis that the effects of COVID‐19 are subsector‐specific. Further research should be undertaken to investigate the causes of these differences. Were the differences between the sub‐sectors merely the result of different levels of shocks caused by lockdowns and social distancing, or are some sub‐sectors also more resilient?

The decline in volatility in April (Figure 2) suggests that investors were slightly more optimistic about stock price developments than in March. In contrast, some companies have withdrawn their outlook, pointing out the many imponderables that make it difficult to forecast. The financial reports, however, offer additional information that was not considered in this study. First, companies describe how COVID‐19 has affected their daily business. Second, the presentation of risk factors provides information on how the pandemic might affect companies in the future and which preventive measures they have taken (e.g., increase in liquidity). The information from financial reports can help to describe the impact of the pandemic on businesses in more detail. This can contribute to designing targeted measures for dealing with future shocks.

Surprisingly, the analysis of stock price reactions during the different phases of the pandemic did not show a clear picture of the drivers. Contrary to Ramelli and Wagner (2020), we found a positive and statistically significant effect of profitability in the outbreak phase. This result may be explained by the fact that investors perceived profitable companies as more resilient. Similar to their results, we observed a negative influence of the market beta on returns in the fever phase. Stocks that were riskier than the market received higher discounts from investors and thus lower cumulative returns. Our results also show that debt led to lower returns in the fever and below peak phases. This could indicate the importance of liquidity in times of crisis. In the below peak phase, which Ramelli and Wagner (2020) did not investigate, a high book‐to‐market ratio is negatively associated with cumulative returns. This result may be explained by the fact that a high book‐to‐market value is associated with less flexibility in cutting capital (Zhang, 2005). In addition, we found regional differences in stock price reactions. This could be due to the different containment measures and their timing as well as the interaction with spatial differences in infection rates. Another possible explanation is that financial systems and firm characteristics differ across the three regions (Griffin, 2002; Semenov, 2006).

Although the models explain up to 75 percent of the variance, the results do not always correspond to the theoretical predictions of the financial market models (CAPM, Fama‐French three factor model). Betas greater than one were associated with reductions in cumulative returns in the fever and below peak phase. In the below peak phase, lower market capitalization and high book‐to‐market value resulted in lower returns, contrary to the predictions of the Fama‐French three factor model. These deviations may be explained by the fact that investors evaluate risks differently in times of crisis (see also Zhang, 2005).

Policy makers can use our findings as a first indication of the effects of COVID‐19, to identify weaknesses in the food supply chain that could endanger the continuity of food supply. Although fertilizers and agrochemicals are not everyday products, price fluctuations can affect input prices for agriculture and thus affect the entire supply chain. If a company is particularly vulnerable to shocks of this kind, diversification may be helpful in buffering future shocks. Many stock‐listed companies are already diversified. However, due to the classification into sub‐sectors, this could not be taken into account in the analysis. Our results give first indications for further research questions. They suggest that the position in the supply chain influences how strongly the shock affects the stock price of companies. Further research could verify this assumption and also include key figures on the length and complexity of supply chains. Additional potential for further analyses results from the time limit of our data set. At a later date, it will be possible to provide more precise information on how severely companies were affected. This would also contribute to the question of how well a short‐term view of stock prices can predict the long‐term consequences of a shock.

By examining large stock‐listed companies, we were able to approximate the short and long‐term effects of the pandemic on the selected companies in the food supply chain. However, with a small sample size, caution must be applied, as the findings might not be representative for other companies. This caution is particularly important in view of the large number of small and medium‐sized businesses in the supply chain as well as the importance of co‐operatives, as these companies are typically not listed on the stock exchange. Both the impact of the pandemic on these companies and their ability to respond to it may differ from stock‐listed companies. Nevertheless, based in the market shares of the companies investigated, we think that our findings are of practical relevance. An additional note of caution is due here since market betas are not stable over time (Groenewold & Fraser, 1999); our betas can therefore be seen as proxies. Notwithstanding these limitations, this study can be seen a first step towards understanding the drivers of stock returns of firms in food supply chains in periods that are preceded by a large shock.

## Data Availability

The data that support the findings of this study are available from the corresponding author upon reasonable request.

## Footnotes

1 Both studies define agribusinesses as companies that support farm production and the conversion of agricultural products to consumable goods.

## APPENDIX A OVERVIEW OF COMPANIES IN THE DATA SET BY SUBSECTOR

**Table:**

**Subsector**	**Company**	**Index**
Agricultural and farm machinery	Deere & Co.	S&P 500
	Kubota	Nikkei225
Agricultural products	Archer‐Daniels‐Midland Co	S&P 500
	Nisshin Seifun Group	Nikkei225
Brewers	AB InBev	BEL
	Asahi Group Holdings	Nikkei225
	Heineken	AEX
	Kirin Holdings	Nikkei225
	Molson Coors Brewing Company	S&P 500
	Sapporo Holdings	Nikkei225
	Takara Holdings	Nikkei225
Distillers and vintners	Brown‐Forman Corp.	S&P 500
	Constellation Brands	S&P 500
	Diageo	FTSE 100
	Pernod Ricard	CAC40
Fertilizers and agricultural chemicals	BASF	DAX
	Bayer	DAX
	CF Industries Holdings Inc	S&P 500
	Corteva	S&P 500
	FMC Corporation	S&P 500
	Sumitomo Chemical	Nikkei225
	The Mosaic Company	S&P 500
Food distributors	Marubeni	Nikkei225
	Sojitz	Nikkei225
	Sysco Corp.	S&P 500
Food retail	AEON	Nikkei225
	Ahold Delhaize	AEX
	Carrefour	CAC40
	Colruyt	BEL
	Familymart Uny Holdings	Nikkei225
	Isetan Mitsukoshi Holdings	Nikkei225
	J. Front Retailing	Nikkei225
	Kroger Co.	S&P 500
	Morrisons	FTSE 100
	Ocado	FTSE 100
	Sainsbury's	FTSE 100
	Seven & i Holdings	Nikkei225
	Tesco	FTSE 100
Hypermarkets and super centers	Costco Wholesale Corp.	S&P 500
	Walmart	S&P 500
Packaged foods and meats	Ajinomoto	Nikkei225
	Associated British Foods	FTSE 100
	Campbell Soup	S&P 500
	Conagra Brands	S&P 500
	Danone	CAC40
	General Mills	S&P 500
	Hormel Foods Corp.	S&P 500
	JM Smucker	S&P 500
	Kellogg Co.	S&P 500
	Kikkoman	Nikkei225
	Kraft Heinz Co	S&P 500
	Lamb Weston Holdings Inc	S&P 500
	McCormick & Co.	S&P 500
	Meiji Holdings	Nikkei225
	Mondelez International	S&P 500
	Nestlé	smi
	Nh Foods	Nikkei225
	Nichirei	Nikkei225
	Nippon Suisan Kaisha	Nikkei225
	The Hershey Company	S&P 500
	Tyson Foods	S&P 500
	Unilever	AEX
	Unilever	FTSE 100
Soft drinks	Coca‐Cola Company	S&P 500
	Coca‐Cola HBC	FTSE 100
	Monster Beverage	S&P 500
	PepsiCo Inc.	S&P 500
Tobacco	Altria Group Inc	S&P 500
	British American Tobacco	FTSE 100
	Imperial Brands	FTSE 100
	Philip Morris International	S&P 500

## APPENDIX B ROBUSTNESS CHECK WITH 2019 DATA

**Table:**

	Dependent variable: Cumulative returns				
	Year 2019	Incubation	Outbreak	Fever	Below Peak
		January 2–17	January 20–February 21	February 24–March 20	March 23–April 29
	(1)	(2)	(3)	(4)	(5)
Beta	4.293	−0.354	−3.376	−34.107***	−23.460***
	(8.385)	(1.701)	(2.985)	(8.623)	(6.746)
Log market capitalization 2019	2.283**	0.003	‐0.201	0.377	0.317
	(0.895)	(0.182)	(0.319)	(0.921)	(0.72)
Profitability 2019	0.367	0.019	0.258	−0.955*	−0.626
	(0.52)	(0.105)	(0.185)	(0.535)	(0.418)
Book‐to‐market 2019	−17.619**	−2.871*	−0.271	2.255	0.525
	(7.620)	(1.545)	(2.712)	(7.836)	(6.130)
Cash/assets 2019	−0.454	0.168	0.102	0.389	0.343
	(0.504)	(0.102)	(0.179)	(0.518)	(0.405)
Leverage 2019	0.014	0.031	−0.009	−0.473**	−0.266*
	(0.181)	(0.037)	(0.065)	(0.187)	(0.146)
Dummy Japan	−4.13	3.333*	−5.635*	−7.954	−16.500**
	(8.572)	(1.739)	(3.051)	(8.815)	(6.897)
Dummy US	1.084	1.159	1.2	7.705	11.177**
	(5.550)	(1.126)	(1.975)	(5.707)	(4.465)
Observations	56	56	56	56	56
*R* ^2^	0.614	0.376	0.291	0.762	0.621

*Note*: **p* < .1; ***p* < .05; ****p* < .01.
